# Eotaxin-1/CCL11 promotes cellular senescence in human-derived fibroblasts through pro-oxidant and pro-inflammatory pathways

**DOI:** 10.3389/fimmu.2023.1243537

**Published:** 2023-10-04

**Authors:** Patrícia Lavandoski, Vinícius Pierdoná, Rafael Moura Maurmann, Lucas Kich Grun, Fatima T. C. R. Guma, Florencia María Barbé-Tuana

**Affiliations:** ^1^ Programa de Pós-Graduação em Ciências Biológicas, Bioquímica do Departamento de Bioquímica, Instituto de Ciências Básicas da Saúde da Universidade Federal do Rio Grande do Sul, Porto Alegre, Rio Grande do Sul, Brazil; ^2^ Programa de Pós-Graduação em Biologia Celular e Molecular da Escola de Ciências da Saúde e da Vida - Pontifícia Universidade Católica do Rio Grande do Sul, Porto Alegre, Rio Grande do Sul, Brazil; ^3^ Programa de Pós-Graduação em Pediatria e Saúde de Criança da Escola de Medicina, Pontifícia Universidade Católica do Rio Grande do Sul, Porto Alegre, Rio Grande do Sul, Brazil

**Keywords:** CCL11, eotaxin-1, senescence, fibroblasts, asthma, premature aging, lung

## Abstract

**Introduction:**

Eotaxin-1/CCL11 is a pivotal chemokine crucial for eosinophil homing to the lungs of asthmatic patients. Recent studies also suggest that CCL11 is involved in the aging process, as it is upregulated in elderly, and correlated with shorter telomere length in leukocytes from asthmatic children. Despite its potential pro-aging effects, the precise contribution of CCL11 and the underlying mechanisms involved in the promotion of cellular senescence remains unclear. Therefore, the primary goal of this study was to explore the role of CCL11 on senescence development and the signaling pathways activated by this chemokine in lung fibroblasts.

**Methods:**

To investigate the targets potentially modulated by CCL11, we performed an *in silico* analysis using PseudoCell. We validated *in vitro* the activation of these targets in the human lung fibroblast cell line MRC-5 following rhCCL11 exposure. Finally, we performed differential gene expression analysis in human airway epithelial cells of asthmatic patients to assess CCL11 signaling and activation of additional senescent markers.

**Results:**

Our study revealed that eotaxin-1/CCL11 promote reactive oxygen secretion (ROS) production in lung fibroblasts, accompanied by increased activation of the DNA damage response (DDR) and p-TP53 and γH2AX. These modifications were accompanied by cellular senescence promotion and increased secretion of senescence-associated secretory phenotype inflammatory cytokines IL-6 and IL-8. Furthermore, our data show that airway epithelial lung cells from atopic asthmatic patients overexpress CCL11 along with aging markers such as CDKN2A (p16INK4a) and SERPINE1.

**Discussion:**

These findings provide new insights into the mechanisms underlying the pro-aging effects of CCL11 in the lungs of asthmatic patients. Understanding the role of CCL11 on senescence development may have important implications for the treatment of age-related lung diseases, such as asthma.

## 1 Introduction

Asthma is a chronic noncommunicable inflammatory disease characterized by bronchial hyperresponsiveness, airway obstruction, increased mucus secretion, chronic airway inflammation, and extracellular matrix remodeling (1). Asthma symptoms may include shortness of breath, chest tightness, cough, and expiratory wheezing on auscultation (2). This condition affected around 262 million people worldwide in 2019, causing 455.000 deaths (3), and represents a major burden to patients and health systems around the world.

Asthma is a heterogeneous disease with various endotypes, and the most common one is type-2 inflammation, affecting more than 80% of children and around 50% of asthmatic adults (1, 2). This inflammatory response is orchestrated by lymphocytes in response to common allergens. During the sensitization phase, allergen-specific CD4^+^ T helper 2 (TH2) cells infiltrate and produce type-2 cytokines, primarily interleukin (IL)-4, IL-5, IL-9, and IL-13 (4, 5). While eosinophils constitute the majority of the inflammatory infiltrate in type-2 asthmatic patients, mast cells, neutrophils, TH2 lymphocytes, and monocytes/macrophages are also present (6–8), illustrating the complex inflammatory nature of asthma.

In addition to type-2 cytokines, epithelial cells play a crucial role by secreting eotaxin-1/CCL11, a central chemoattractant for eosinophil homing to the lungs. This chemokine is observed in asthmatic patients’ bronchial mucosal biopsy in correlation with increased susceptibility to exacerbation (9) and is also increased in plasma and sputum from atopic children (10, 11).

Furthermore, CCL11 appears to be involved in lung fibrosis, a central feature for tissue remodeling observed in asthmatic patients, as its depletion prevented pulmonary fibrosis in mice (12). Additionally, CCL11 promotes proliferation and increased collagen expression in lung fibroblasts, suggesting its role in extracellular matrix deposition and tissue remodeling in asthma mediated by fibroblasts (13).

Interestingly, CCL11 has been recognized as a regulator of physiological decline in chronological aging. Plasmatic concentrations of CCL11 are increased in older subjects (14, 15) and associated with systemic aging, poor cognitive function, and decreased neurogenesis (15). Moreover, elevated levels of CCL11 are observed in severe asthmatic children and correlated with reduced telomere length in peripheral blood mononuclear cells (PBMC), an important marker of cellular aging (11).

A central feature of aging is the increased prevalence of senescent cells in different tissues, leading to decreased function and ultimately to organ failure (16). Cellular senescence is defined as an irreversible cell cycle arrest even in the presence of mitogenic factors and represents the aging process at cellular level (17, 18).

Senescence can be triggered by persistent DNA damage signals, leading to the stabilization of tumor protein P53 (TP53) and induction of cyclin-dependent kinase inhibitors, such as P21 (CDKN1A), and P16 (p16INK4a, CDKN2A). Additionally, senescence is associated with phenotypic modifications, including augmented cell size and nuclear area, increased β-galactosidase activity (19), reactive oxygen species (ROS) overproduction, and the secretion of inflammatory proteins and matrix metalloproteinases, which constitute the senescence-associated secretory phenotype (SASP) (20, 21).

During chronological aging, the respiratory tract undergoes several structural and functional changes, such as decreased lung function, declining maximal aerobic capacity, reduced pulmonary forced expiratory volume, tissue remodeling, compromised regeneration, and enhanced susceptibility to respiratory infections (22). Furthermore, key senescence mediators such as TP53, P21, and the phosphorylated histone H2AX (γH2AX), an early cell response to DNA double-strand breaks (DSB), are upregulated in aged lungs. These senescence-related alterations play a crucial role in the deterioration of lung function during chronological aging (23).

Aging lungs often experience compromised function due to the establishment of a profibrotic environment with increased extracellular matrix (ECM) deposition, resulting from heightened fibroblast activation. The increased density of lung senescent cells appears to directly contribute to aging-associated ECM changes (23). Considering its central role in orchestrating ECM homeostasis, senescent fibroblasts emerge as crucial drivers of tissue remodeling and potentially contribute to the predisposition to age-related pulmonary diseases, such as chronic obstructive pulmonary disease (COPD) (24, 25). Also, senescent fibroblasts secrete SASP components, such as IL-6 and IL-8, contributing to the chronic pro-inflammatory environment described as a central feature of lung aging (24, 26).

Interestingly, many of these aging features, such as decreased lung function and airway tissue remodeling are shared by asthmatic patients (1). Therefore, it is possible that the lung parenchyma in asthma undergoes a process of premature aging due to increased stress caused by inflammation and ROS overproduction, which may result from fibroblast dysfunction (27). In this report, our main goal was to investigate whether CCL11, a prominent pro-aging chemokine, is capable of inducing senescence in human-derived fibroblasts and the role of oxidative stress and inflammation in this process. Our results demonstrate that CCL11 induced ROS and cytokine overproduction, leading to cellular senescence in human-derived lung fibroblasts.

## 2 Materials and methods

### 2.1 Cell culture

MRC5 is a diploid fibroblast cell line isolated from lung tissue of a white, male, 14-week-old human embryo (HEF). MRC5 cell line was purchased from American Type Culture Collection (ATCC, CCL-171™, Rockefeller, MD, USA). Cells were cultured in low-glucose (1 g/L) Dulbecco’s Modified Eagle’s Medium (Gibco™, Waltham, USA), supplemented with 10% fetal bovine serum (Gibco), and 1% (v/v) penicillin-streptomycin (10,000 units penicillin and 10 mg streptomycin/mL, Sigma-Aldrich®, San Luis, USA) at 37°C in 5% CO_2_ humidified atmosphere incubator. When necessary, cells were detached with trypsin (0.05% trypsin-EDTA 1:10 in saline, Gibco), inactivated with 2:1 of DMEM low glucose (Gibco) supplemented with 10% FBS and used for subsequent analyses.

### 2.2 Treatment with rhCCL11

Recombinant human CCL11 (rhCCL11, PeproTech, Waltham, USA) was reconstituted in phosphate buffered saline supplemented with 0.18g/100 mL of glucose, aliquoted and frozen at -80°C until use. MRC5 cells were seeded at 10^5^/mL and treated with increasing concentrations of rhCCL11 as described for each assay.

### 2.3 Evaluation of reactive oxygen species production

Intracellular reactive oxygen species (ROS) production was evaluated using 2′-7′-dichlorofluorescein diacetate (DCFH-DA, Sigma-Aldrich). MRC5 cells were cultured in 96 well plate (10^4^ cells/200uL final solution) and treated for 4 or 24 hours with 100, 250 and 500ng/10^5^cells/mL doses of rhCCL11. Cells were resuspended in a rhCCL11 solution diluted in Hanks’ Balanced Salt Solution (HBSS) (Sigma-Aldrich) without phenol red. Immediately, cells were incubated in the dark with 10μM DCFH-DA for ROS quantification. Alternatively, cells were treated with increasing concentrations of rhCCL11 in DMEM low glucose (1 g/L) supplemented with 10% FBS (Gibco) 24 hours before ROS quantification. The fluorescence generated by DCF was measured using a 96-well microplate reader (VICTORX Multilabel Plate Readers, PerkinElmer) at 485/535nm (excitation/emission). One hundred twenty (120) readings were performed with 1-second intervals between each measure in the course of 4 hours. The results are expressed as relative fluorescence units (RFU).

### 2.4 Intracellular γH2AX, p-TP53 and Ki-67

MRC5 cells were seeded in a 24 well plate until reach approximately a 10^5^ cells/mL density and then treated with 500ng/10^5^cells/mL of rhCCL11 for 2h or 24h. Cells were trypsinized and washed twice with FACS buffer (saline supplemented with 2% of FBS), fixed with BD Cytofix™ Fixation Buffer (BD Biosciences, San Jose, California) for 20 minutes at 4°C and permeabilized with BD Phosflow™ Perm Buffer III (BD Biosciences) for 30 minutes at 4°C. Cells were washed again with FACS Buffer and incubated with the following antibodies: Alexa Fluor® 647 Mouse anti-H2AX (pS139) 1:200 (#560447 BD Biosciences) and Alexa Fluor® 488 Mouse anti-p53 (pS37) 1:50 (#560282 BD Biosciences) for 30 minutes at 4°C on dark. For the proliferation assay cells were stained with Alexa Fluor® 488 Mouse anti-Ki-67 1:200 (#561165, BD Biosciences). Finally, cells were washed and resuspended in FACS buffer for acquisition (10.000 events).

### 2.5 Senescence-associated-β-galactosidase activity

MRC5 cells were seeded in a 24 well plate until reach approximately a 10^5^ cells/mL density and then treated with 500ng/10^5^cells/mL of rhCCL11 for 24 hours or 50ng/mL for 5 days in DMEM low glucose (1 g/L) supplemented with 10% FBS. For the 5-day treatment, MRC5 cells were exposed at day 1 to 50 ng/mL of rhCCL11. After 3 days, 50% of the media was replaced with fresh DMEM low glucose 10% FBS media containing rhCCL11 to achieve a final concentration of 50 ng/mL. At day 5, the cells were trypsinized and assessed for C_12_FDG activity. Senescence-associated β-galactosidase (SA-β-gal) activity was evaluated using the fluorogenic substrate C12FDG (5-Dodecanoylaminofluorescein Di-β-D-Galactopyranoside, Invitrogen™). For SA-β-gal activity, cells were trypsinized and treated with 100µM of chloroquine for 1h at 37°C and 5% CO_2_ humidified atmosphere to induce lysosomal alkalinization. Then, C12FDG (33μM) was added, and cells were incubated for an additional 1h before acquisitions by flow cytometry.

### 2.6 MTT Assay

Cell viability was measured by MTT (3-4,5-dimethylthiazolyl-2,5-diphenyl-2H-tetrazolium bromide, Sigma Inc.). MRC5 were cultured in 96 well plate (10^4^ cells/well) and incubated with increasing concentrations of rhCCL11 in DMEM low glucose 10% FBS (Gibco) for 4 hours as described on section 2.3. For formazan crystal formation quantification, cells were incubated with 0.5mg/mL of MTT for 2 h at 37°C, lysed in dimethyl sulfoxide (DMSO, Sigma-Aldrich) and read at absorbance λ = 570 nm in a 96-well microplate spectrophotometer (Zenyth 340r Microplate Reader) with previous agitation.

### 2.7 Cytometric bead array

Cytokine secretion was measured in cell culture media 24 hours after treatment with 500ng/10^5^cells/mL of rhCCL11 by Cytometric Bead Array with the Human Inflammatory Kit (BD Biosciences) according to the manufacturer’s instructions. Data was analyzed with FCAP Array v3.0.1 software (Soft Flow Inc., Pecs, Hungary). Standard curves were run in duplicate, and results were expressed as picograms per milliliter (pg/mL). Theoretical lower limits of detection accordingly to the manufacturer are IL-8 (3.6 pg/mL), IL-1β (7.2 pg/mL), IL-6 (2.5 pg/mL), IL-10 (3.3 pg/mL), TNF-α (3.7 pg/mL) and IL-12p70 (1.9 pg/mL). When analyzing cytokine levels, samples with values below the lower limit of detection (LLOD) were assigned a value of LLOD divided by the square root of two. Samples with values between the LLOD and upper limit of detection (ULOD) were considered detectable for specific cytokines (28).

All acquisitions were made in a BD FACSCanto™ II (BD Biosciences) with a 3-laser, 4-2-2 configuration (Violet 405 nm, Blue 488 nm and Red 633 nm). FACS data from immunophenotyping was analyzed with FlowJo™ v10.8.1 (BD Life Sciences).

### 2.8 ELISA assay

MRC-5 cells were seeded in 24-well plate at 1x10^5^ cells/well and treated with 500ng/10^5^cells/mL of rhCCL11 for 24 hours. Each well was then washed with saline, reconstituted with new media (DMEM low glucose 10% FBS) and incubated for another 24 hours at 37°C and 5% CO_2_ humidified atmosphere. Eotaxin-1/CCL11 levels were measured by enzyme-linked immunosorbent assay (ELISA) (Human Eotaxin (CCL11) Standard ABTS ELISA Development Kit, Peprotech) according to the manufacturer’s instructions from 100µL of conditioned media stored at -80° C.

### 2.9 *In silico* simulation of increasing CCL11 frequency of activation

PseudoCell is a software application that was developed to enable *in silico* prediction of molecular interactions between proteins and metabolites, referred to as nodes. The tool provides users with the capability to assess the dynamics of a network’s response to disturbances or stimuli applied to a designated target over time (29). In this study, we employed PseudoCell to emulate the behavior of a cell in response to increasing concentrations of CCL11. To this end, we subjected the CCL11 node to various frequencies of activation, including 0% (control), 12.5% (low expression), 25% (medium expression), and 50% (high expression). The network was then updated for 1,000 cycles, and the simulations were repeated 30 times for each experimental group (n=30).

### 2.10 Differential gene expression (DGE) and gene ontology (GO) enrichment analysis

Differential gene expression analysis (DGE) was performed using a microarray dataset (GSE18965) (28) through the GEO2R open-source interactive web tool. Were considered differentially expressed (DE) those genes with p <0.05 after Benjamini & Hochberg (False discovery rate, FDR) correction (29). The gene ontology (GO) enrichment analysis for biological process and molecular function were performed for targets that remained differentially expressed after Benjamini & Hochberg FDR correction through GO Consortium (30–32).

### 2.11 Statistics

Statistical analysis was performed with GraphPad Prism 6.01 (GraphPad Software Inc., LaJolla, California). Sample distribution of each group was initially assessed using the Shapiro-Wilk normality test. For comparisons between two groups, the Student t-test was employed for samples with a normal distribution, whereas the Mann-Whitney U-test was used for non-parametric samples. For analyses involving three or more groups, the Ordinary One-way ANOVA followed by Dunnett’s multiple comparison test was performed for samples with a normal distribution, while the Kruskal-Wallis test followed by Dunn’s multiple comparisons test was employed for non-parametric samples. The presented data are expressed as median and Interquartile Range (IQR), as specified in each figure’s description. Significant differences were considered when P<0.05.

## 3 Results

### 3.1 *In silico* stimulation of CCL11 directly promotes oxidative stress, DNA damage and pro-inflammatory pathways

PseudoCell is an open-source regulatory network capable of robustly simulating intricate molecular networks and forecasting potential outcomes following disturbances to specific targets (29). In this study, we employed PseudoCell as a guiding tool for hypothesis formation, enabling us to identify and evaluate the most probable targets that undergo perturbations upon exposure to CCL11 in a complex biological system.

**Figure 1:**
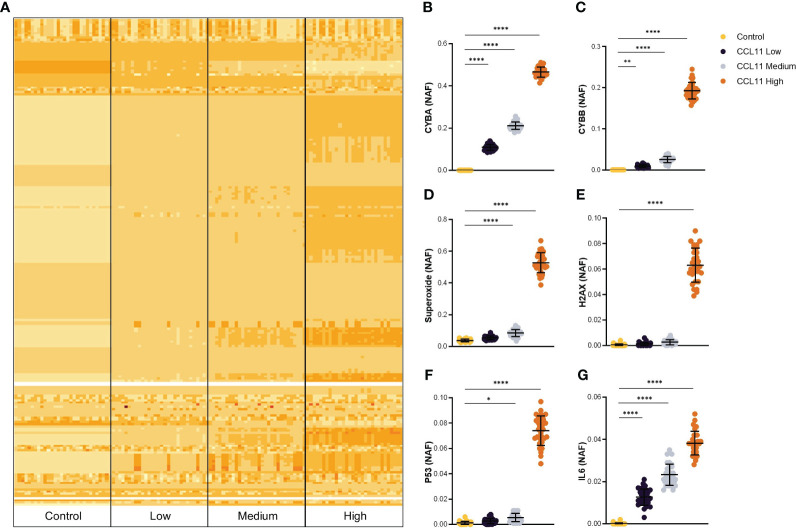
*In silico* prediction of potential targets activated after CCL11 stimuli. **(A)** Heatmap plot representing alteration in activation frequencies patterns for Control, CCL11 Low, CCL11 Medium and CCL11 High groups. Augmented Node Activation Frequency (NAF) for CYBA **(B)**, CYBB **(C)**, Superoxide **(D)**, H2AX **(E)**, TP53 **(F)** and IL6 **(G)** induced by increased *in silico* activation of CCL11. **(B–D)** Ordinary One-way ANOVA followed by Dunnett’s multiple comparison test was performed. **(E–G)** Kruskal-Wallis test followed by Dunn’s multiple comparisons test was performed. Data are presented as median and IQR and display 30 independent *in silico* experiments. Significant differences considered when P<0.05 (*), P<0.01 (**) and P<0.0001 (****).

Upon challenging the PseudoCell software with increasing activation frequencies of CCL11, we observed a notable alteration in the network behavior, mainly at the “CCL11 High” group, as demonstrated by the heatmap plot presented in **Figure 1A**. Notably, increasing Node Activation Frequencies (NAF) of CCL11 induced a dose-dependent overexpression of cytochrome b alpha (CYBA) and beta (CYBB) chains of the protein Cytochrome b-245, responsible for ROS formation (**Figures 1B, C**). These findings were further supported by the overexpression of superoxide at the medium and high CCL11 stimuli conditions (**Figure 1D**). In addition, we observed that the highest stimuli frequency induced remarkable activation of two important proteins involved in DNA damage signaling, namely the H2A Histone Family Member X (H2AX) and the tumor suppressor protein TP53 (**Figures 1E, F**). Finally, the CCL11 activation led to a dose-dependent increase in IL6, an essential pro-inflammatory cytokine that is known to increase during aging and composes the senescence-associated secretory phenotype (SASP) (**Figure 1G**).

### 3.2 CCL11 induces ROS production in fibroblasts

**Figure 2:**
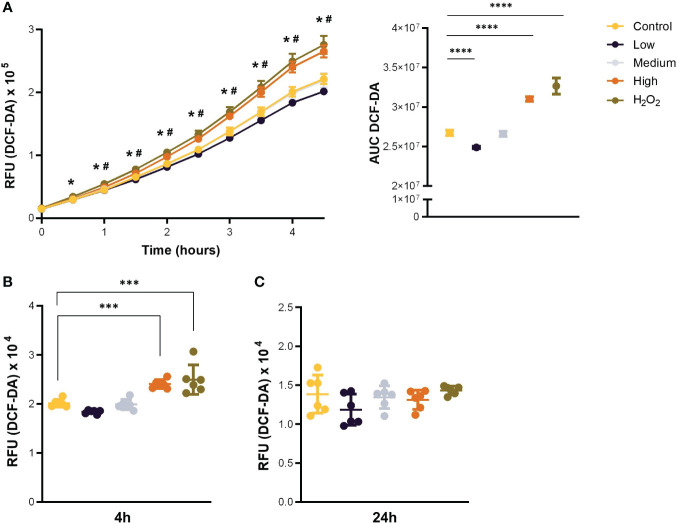
Acute stimulation with CCL11 induces ROS overproduction. MRC-5 cells were incubated with low, medium, and high doses (100, 250 and 500ng/10^5^ cells/mL, respectively) of rhCCL11 for 4 hours or 24 hours. **(A)** Increased ROS generation after exposure to rhCCL11 high. Data presented as relative fluorescence units (RFU) emission over time (left), and Area Under the Curve (AUC) (right). Significant differences are shown between Control vs H_2_O_2_ (*) and Control vs CCL11 High (#). **(B)** Increased ROS production 4 hours after treatment with rhCCL11 high and **(C)** no differences between groups with a 24-hour exposure to rhCCL11. Ordinary One-way ANOVA followed by Dunnett’s multiple comparison test was performed for all experiments. Data are presented as median and IQR. N = 6 for all groups. Significant differences considered when P<0.05 (*), P<0.001 (***) and P<0.0001 (****).

Given that a hallmark of stress-induced senescence is a shift towards a pro-oxidative phenotype, and our model predicted an increase in oxidative stress after exposure to CCL11, we aimed to validate these results *in vitro*. To this end, we treated the MRC5 human-derived fibroblast cell line with increasing doses of recombinant human CCL11 (rhCCL11) and assessed ROS formation. Our results showed that treatment with the highest dose of rhCCL11 induced overproduction of ROS within 60 minutes, persisting for up to 4 hours (**Figures 2A, B**) (p<0.05). However, no significant differences were observed in ROS secretion 24 hours after treatment (**Figure 2C**).

### 3.3 Increased DNA damage signaling induced by CCL11

**Figure 3:**
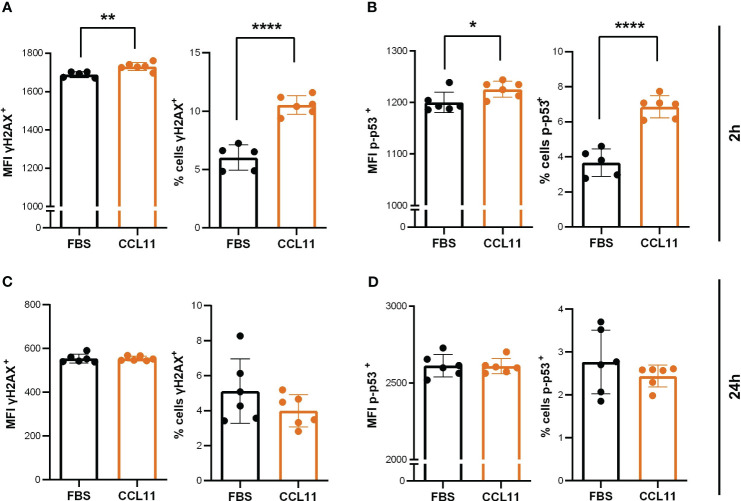
CCL11 induces DNA damage mediated by γH2AX and p-TP53. MRC-5 cells were seeded at 10x10^4^cells/mL and incubated with high doses (500ng/10^5^ cells/mL) of rhCCL11 for 2h or 24h. We observed increased γH2AX **(A)** (N = 5/6, respectively) and TP53 **(B)** (N = 5/6) phosphorylation and activation 2h after exposure to rhCCL11. No differences between groups were observed for γH2AX **(C)** (N = 6) or TP53 **(D)** (N = 6) phosphorylation 24h after treatment. **(A–D)** Unpaired t-test for normal distribution was performed, except for % of cells p53^+^ data **(D)**, for which Mann-Whitney test was performed. Data are presented as median and IQR. Significant differences considered when P<0.05 (*), P<0.01 (**) and P<0.0001 (****). MFI, median fluorescence intensity.

To examine whether the pro-oxidative environment observed in MRC5 after treatment with CCL11 could induce DNA damage signaling, we quantified the phosphorylated active forms of H2AX (γH2AX) and TP53 (phospho-TP53). Our results showed that 2 hours after treatment, there was a significant increase in the percentage (p < 0.0001) and mean fluorescence intensity (MFI) (p = 0.005) of γH2AX-positive cells, as well as an increase in the percentage (p < 0.0001) and MFI (p = 0.0352) of p-p53 expressing cells (**Figures 3A, B**). These findings suggest that CCL11 can activate DNA damage signaling pathways. Interestingly, the DNA damage response pathways were abrogated 24 hours after treatment, implying that the machinery for DNA repair was able to restore cellular homeostasis (**Figures 3C, D**).

### 3.4 CCL11 induces cellular senescence in healthy fibroblasts

**Figure 4:**
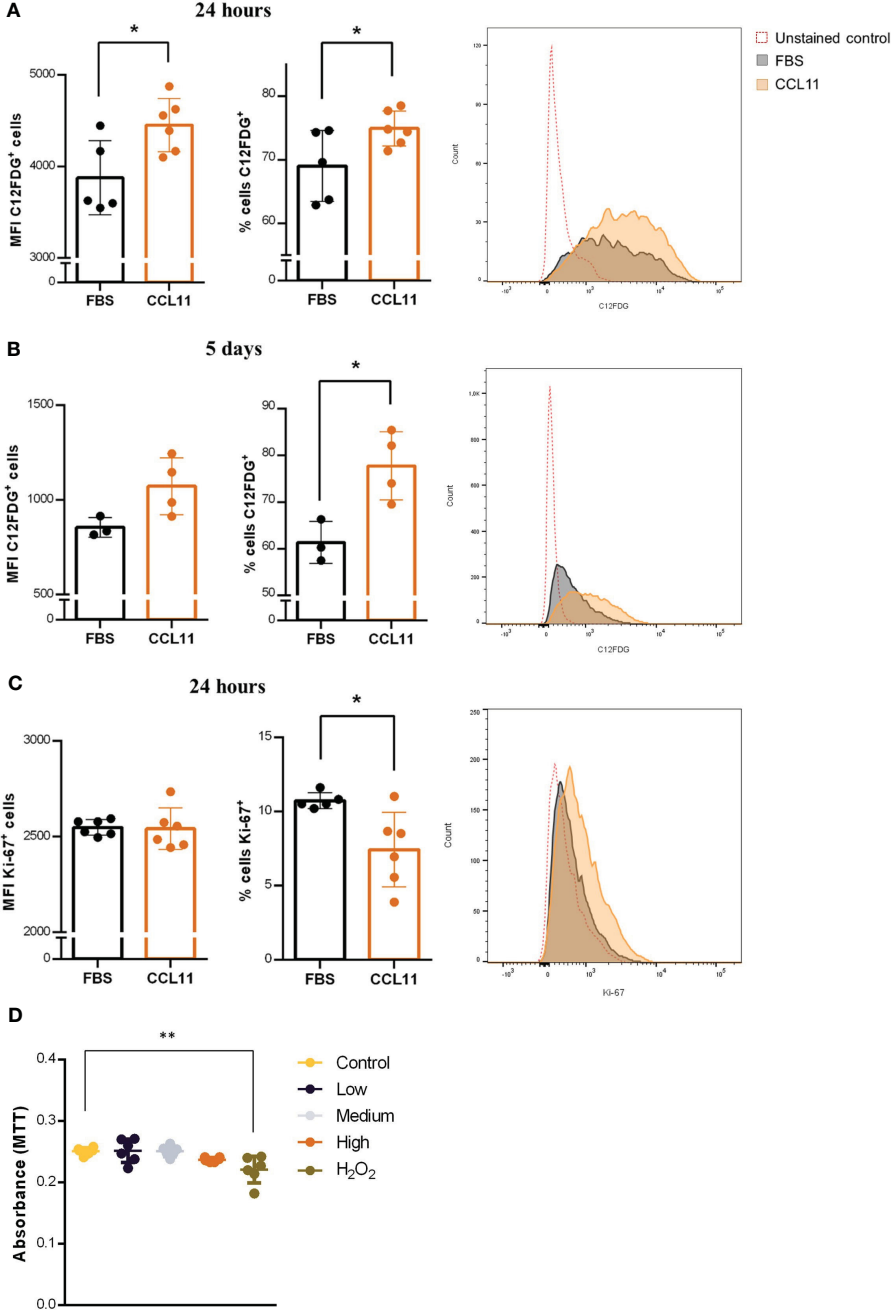
Cellular senescence and reduced proliferation induced by CCL11. MRC-5 cells were incubated with high dose (500ng/10^5^ cells/mL) for 24 hours or low dose (50 ng/mL) of rhCCL11 for 5 days. We observed increased SA-β-Gal activity (C12FDG^+^ cells) cells at 24 hours **(A)** (N = 5/6) and 5 days **(B)** (N = 3/4) after exposure to rhCCL11. **(C)** We also observed reduced number of Ki-67^+^ proliferating cells after the 24-hour treatment (N = 6). **(D)** No differences in cell viability were observed between the groups exposed to CCL11 (N=6). **(A–C)** Unpaired t-test for normal distribution was performed. **(D)** Ordinary One-way ANOVA followed by Dunnett’s multiple comparison test. Data are presented as median and IQR. Significant differences considered when P<0.05 (*) or P<0.01 (**).

Since senescent fibroblasts are involved in deleterious alterations in lung diseases, we sought to evaluate the impact of CCL11 on cellular senescence in the MRC5 human-derived fibroblast cell line. Our results indicate that the 24 hour-treatment with rhCCL11 led to an increase in percentage (p = 0.0481) and MFI (p = 0.0229) of SA-β-gal activity, a widely accepted biomarker of cellular senescence (**Figure 4A**). Moreover, our findings suggest that even low concentrations of CCL11 can sustain features of cellular senescence after chronic exposure, as evidenced by a similar response in SA-β-gal activity after a 5-day treatment with a ten-times lower dose of rhCCL11 (**Figure 4B**) (p = 0.0194).

Additionally, we observed decrease cell proliferation, represented by reduced Ki-67 expression, in fibroblasts treated with rhCCL11 (**Figure 4C**) (p = 0.0192), another important hallmark of cellular senescence. Notably, there were no significant differences in cell viability between groups 4 hours after treatment with rhCCL11. Decreased viability was only present on the positive control group incubated with 100µM H_2_O_2_ (**Figure 4D**) (p = 0.0025). Collectively, our findings provide evidence that CCL11 is capable of inducing cellular senescence in human lung-derived fibroblasts, which may contribute to pathological extracellular matrix alterations in lung diseases.

### 3.5 CCL11 induces a pro-inflammatory loop through SASP secretion

**Figure 5:**
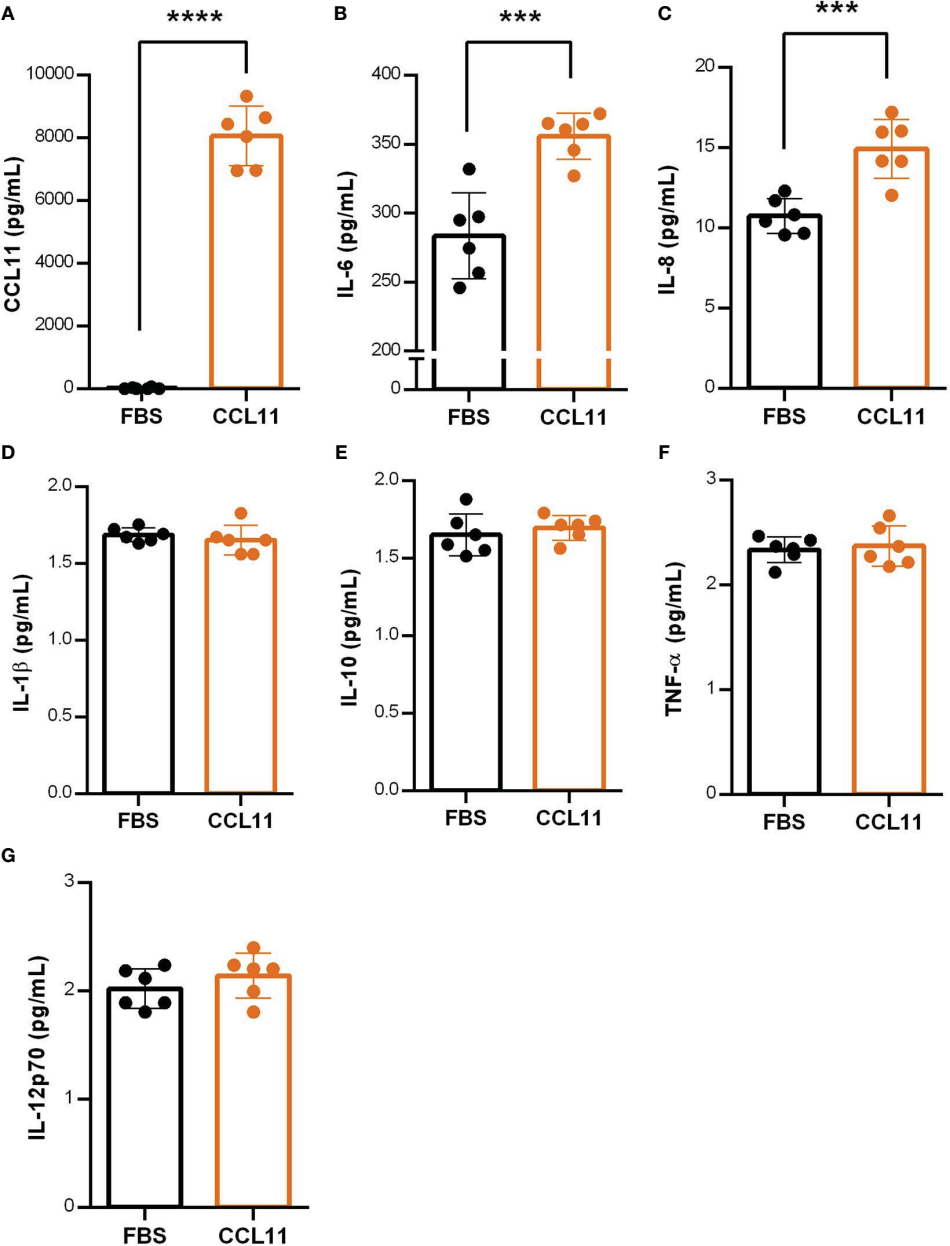
CCL11 induces positive feedback on CCL11 secretion and increases SASP mediators. MRC-5 cells were pre-treated with rhCCL11 (500ng/10^5^ cells/mL) for 24 hours and supernatant was replaced for fresh media without rhCCL11 for additional 24 hours for cytokine secretion quantification. **(A)** Increased CCL11 secretion on cells pre-treated with rhCCL11 evaluated by ELISA. **(B–G)** Increased secretion of the SASP cytokines IL-6 and IL-8 after 24 hours treatment, with no differences observed in the remained cytokines (N = 6). **(A)** Mann-Whitney U-test for non-parametric samples was performed. **(B–G)** Unpaired t-test for normal distribution was performed. Data are presented as median and IQR. Significant differences considered when P<0.001 (***) or P<0.0001 (****).

To deeper understand the mechanisms underlying the mediation of the inflammatory process by eotaxin-1, we investigated the endogenous production of CCL11 by fibroblasts 24 hours after treatment with the recombinant homologous chemokine. Our findings revealed a significant increase in CCL11secretion by fibroblasts pre-treated with CCL11 (**Figure 5A**) (p<0.0001), indicating the presence of a positive feedback loop and an amplification of the pro-senescent microenvironment promoted by CCL11.

Additionally, we aimed to identify pro-inflammatory cytokines recognized as components of the SASP. Our results showed augmented secretion of IL-6 (p = 0.0005) and IL-8 (p = 0.0008) (**Figures 5B, C**) in fibroblasts treated with CCL11 for 24 hours, suggesting their involvement as key elements in the senescence secretome. No significant differences were observed between groups for the remaining cytokines (**Figures 4D–G**).

### 3.6 Senescent markers and fibroblast survival pathways are observed in lung of asthmatic patients

**Figure 6:**
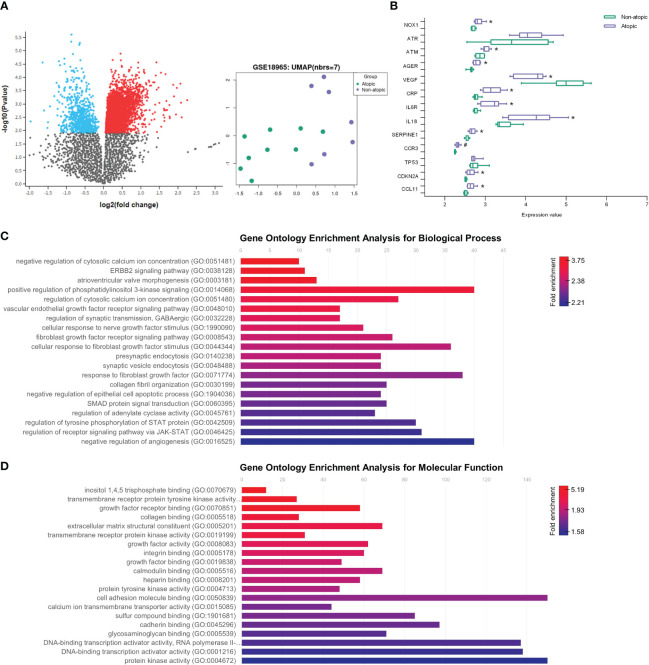
Aging markers and increased CCL11 in airway epithelial cells (AEC) from children with asthma. **(A)** Volcano plot showing increased (red) and decreased (blue) gene expression on airway epithelial cells (AEC) from children with asthma (GSE18965) comparted to atopic controls. Uniform Manifold Approximation and Projection (UMAP) showing two distinguishable classes for asthma and atopic control groups. **(B)** Increased expression of aging markers associated with inflammatory response, oxidative stress, and cellular senescence on AEC from children with asthma (N = 7/9 for healthy controls and atopic asthma, respectively). Unpaired t-test for normal distribution was performed. Data are presented as median and IQR. Enrichment of fibroblast survival and fibrotic pathways on gene ontology (GO) analysis performed for biological process **(C)** and molecular function **(D)** ontology annotations. Significant differences considered when P<0.05 (*) after Benjamini & Hochberg (False discovery rate, FDR) correction or P <0.05 (#) prior to normalization.

To establish a connection between *in silico* and *in vitro* findings with clinical data, we conducted a differential gene expression analysis using a microarray dataset derived from airway epithelial cells (AECs) of children with asthma and healthy non-atopic controls (GSE18965) (**Figure 6A**). Our primary goal was to investigate the role of epithelial cells in inducing fibroblast senescence and to explore the existence of a pro-senescent environment within the lung parenchyma. Specifically, we focused on assessing whether there is an upregulation of CCL11 expression in AEC cells, which could potentially function as the primary source of CCL11 due to their direct interface with the lung tissue and allergens, thereby promoting fibroblast senescence.

Our analysis revealed upregulation of eotaxin-1/CCL11 expression in the airway epithelial cells of asthmatic patients. Additionally, we observed increased expression of NADPH oxidase 1 (NOX1) and ATM serine/threonine kinase (ATM), along with decreased vascular endothelial growth factor A (VEGFA) levels. Furthermore, pro-inflammatory markers such as IL18, IL6R, and C-reactive protein (CRP) exhibited elevated expression. Notably, we also identified increased expression of serpin family E member 1 (SERPINE1) and cyclin-dependent kinase inhibitor 2A (CDKN2A), important mediators of aging (**Figure 6B**). These findings shed light on the molecular alterations within the airway epithelial cells that may contribute to lung fibroblast premature aging in asthma.

We further conducted a GO enrichment analysis to investigate the primary biological processes triggered by asthma in the lungs of asthmatic patients, which led to the identification of several enriched pathways (**Figures 6C, D**). Notably, our analysis revealed a significant enrichment of fibroblast survival-related pathways in atopic asthma subjects, prominently including the fibroblast growth factor receptor signaling pathway. These findings further underscore the close interplay between lung epithelial cells and fibroblast dysfunction in the context of asthma.

## 4 Discussion

In this study, we demonstrated that eotaxin-1/CCL11 was able to induce cellular senescence and sustain a pro-inflammatory response within human embryonic fibroblasts through promotion of oxidative stress-induced DNA damage. Our findings suggest that CCL11 signaling might play a pivotal role in establishing a pro-aging environment in the lungs of asthmatic patients, with resident fibroblasts playing a critical role in this process.

CCL11 is a protein secreted by several cell types in the respiratory tract, including airway epithelial cells, fibroblasts, smooth muscle cells, and macrophages. Its primary function in the airways is to act as a chemoattractant for eosinophils, which can lead to allergen-induced lung eosinophilia (1, 30, 31). Activated eosinophils are a significant source of ROS during asthma exacerbations. Interestingly, ROS production by eosinophils is induced in a dose-dependent manner by CCL11, leading to oxidative damage and lung tissue injury (32, 33). Treating eosinophils with CCR3 antagonists reduces ROS production, emphasizing the role of CCL11 in promoting oxidative stress (33, 34). Our study demonstrates that CCL11 triggers ROS overproduction also in lung-derived fibroblasts, suggesting that this chemokine can promote a pro-oxidative environment by stimulating multiple cellular sources in asthmatic lungs.

At cellular level, ROS can induce DNA oxidative damage, including DNA single-strand breaks (SSBs) or double-strand breaks (DBSs). To prevent the propagation of deleterious mutations, cells employ a complex system known as the DNA damage response (DDR). This system promotes the mobilization of various molecular components to either facilitate DNA damage repair or prevent cell replication by inducing apoptosis or senescence (35).

Upon detection of SSBs or DSBs, the cell orchestrates the recruitment of specific protein kinases such as ataxia telangiectasia and Rad3-related (ATR) and ataxia-telangiectasia mutated (ATM), respectively (36). Upon recruitment to the lesion sites, these kinases trigger the phosphorylation and activation of H2AX, culminating in the formation of γH2AX. This activated variant is translocated to the DNA damage sites amplifying the DDR response (36). Moreover, DNA damage kinases are involved in phosphorylation of TP53, a pivotal regulator of cell cycle arrest and cellular senescence (37).

Notably, our findings support this intricate interplay between ROS overproduction and the promotion of DNA damage in triggering cellular senescence following exposure to CCL11. Although in the present study we did not use inhibitors to support a direct evidence and causal relationship between ROS overproduction and DDR signaling, our findings reveal a simultaneous rise in ROS levels, and H2AX and TP53 activation through phosphorylation, in the lung fibroblast cell line MRC-5 when exposed to CCL11. Therefore, we hypothesize that ROS production may act as a trigger for senescence induction by promoting DNA damage.

Moreover, the enhanced expression of *ATM* in airway epithelial cells of asthmatic children provides additional evidence of DNA damage establishment within the lungs, highlighting its pivotal role as a stress-induced mechanism operating across diverse cell types in the context of asthma.

Additionally, previous studies from our group revealed that ROS depletion by N-Acetyl Cysteine (NAC) partially prevented γH2AX activation induced by CCL11 in PBMC, reinforcing that ROS might play an important role in the mechanism underlying CCL11-induced effects (29). Therefore, it seems that ROS is not only present but essential for the establishment of a persistent DDR leading to premature aging.

Regarding the role of CCL11 in inducing premature aging within the context of asthma, we have shown a decrease in telomere length in peripheral blood mononuclear cells (PBMC) accompanied by an increased expression of eotaxin-1/CCL11 in plasma samples from severe asthma patients. Intriguingly, we also observed an inverse correlation between CCL11 levels and telomere length, implying that high concentrations of this chemokine may lead to telomere shortening (11). These results suggest that CCL11 may contribute to the premature aging in severe asthma patients and highlight the importance to further elucidate the underlying mechanisms involved on this process.

Interestingly, ROS also appears to play a pivotal role in premature senescence mediated by telomere shortening, which also contributes to persistent DDR (38, 39). Previous research has demonstrated a consistent increase in the telomere shortening rate under increased oxidative stress (10). Additionally, antioxidant treatments, such as ascorbic acid 2-O-phosphate and the free-radical scavenger α-phenyl-t-butyl-nitrone, prevented premature aging and extended the lifespan of human endothelial and fibroblast cells (40, 41).

Further supporting the role of CCL11 in promoting cellular senescence, we observed an increased expression of SASP markers, exemplified by augmented IL-6 and IL-8 secretion in fibroblasts exposed to CCL11. These findings are consistent with previous observations that have highlighted IL-6 and IL-8 as the primary cytokines released by senescent fibroblasts undergoing proliferative exhaustion, indicating their pivotal role as mediators of SASP (42). Notably, there are reports pointing out that CCL11 can also induce the secretion of IL-8 and GM-CSF in bronchial epithelial cells, suggesting its ability to promote cytokine upregulation in different populations (43). Furthermore, our data reveals enhanced expression of IL6R and *CRP* in AECs derived from atopic-asthma patients. Interestingly, a positive correlation exists between these inflammatory markers and CCL11 (**Supplementary Figures 1F, G**), implying the potential role of eotaxin-1 as a mediator of the pro-inflammatory *milieu* observed within the lungs of individuals with asthma.

Finally, we aimed to investigate the contribution of epithelial cells with CCL11 production and the extent to which senescence impacts the lung parenchyma. Specifically, we focused on determining whether airway epithelial cells (AECs) exhibit an elevated expression of CCL11, potentially establishing them as a primary source of this cytokine. Additionally, we sought to evaluate the expression of aging markers within AECs, providing insights into the involvement of senescence in asthma-related lung dysfunction.

Our investigations revealed an increase in *CCL11* expression in AEC cells, implying their substantial contribution to CCL11 secretion. Given the strategic location of AECs as intermediaries between lung tissue and allergens, their capacity to induce fibroblast senescence holds significant implications for lung health and function. Furthermore, our observations unveiled the establishment of a pro-senescent *milieu* in AEC of asthmatic patients, as evidenced by elevated expressions of *SERPINE1* and *CDKN2A* (p16INK4a). These findings emphasize the relevance of senescence in the context of asthma, suggesting a potential positive feedback loop between senescent epithelial cells and fibroblasts. This interplay may play a crucial role in the deterioration of lung function.

Moreover, our study further explores the role of CCL11 as a pro-aging factor in the context of asthma, as evidenced by decreased *VEGF* expression in AEC of asthmatic patients. This observation holds particular relevance due to the established link between reduced *VEGF* expression and aging, which is often accompanied by compromised angiogenesis (44, 45). Notably, our investigation also revealed an inverse correlation between *VEGF* expression and *CCL11* levels, further emphasizing the role of this chemokine in the aging process (**Supplementary Figure 1E**).

Furthermore, our results showed an upregulation of *NOX1* expression in AEC from children with asthma, which directly correlates with the increased levels of *CCL11* (**Supplementary Figure 1A**). *NOX1* is responsible for the catalytic transfer of oxygen and superoxide and hydrogen peroxide generation. NOX-family members are described as major cellular sources of ROS, and *NOX1*-derived ROS are involved in many stress-induced outcomes, suggesting that ROS production through *NOX1* could be involved in lung tissue disfunction and senescence (46, 47). Additionally, the heightened expression of *ATM*, a crucial initiator of DDR after DSBs, associated with the positive correlation observed between *ATM* and *CCL11* in AECs (**Supplementary Figure 1C**), further supports the idea that oxidative stress in asthmatic lungs can induce DNA oxidative damage.

In conclusion, our findings illustrate that eotaxin-1/CCL11 has the capability to induce excessive ROS production in lung fibroblasts. This, in turn, is likely to activate various DDR pathways, which might lead to cellular senescence and the amplification of the pro-inflammatory response. Furthermore, our data reveal an increased expression of multiple aging markers within AECs from atopic asthma patients, accompanied by heightened CCL11 secretion. These observations suggest the establishment of a pro-senescent *milieu* that appears to drive a premature aging process in the context of asthma, aggravated by fibroblast dysfunction.

## Data availability statement

The original codes used in the analyses of this study are publicly available. This data can be found here: https://github.com/viniciuspierdona/pseudocell.

## Ethics statement

Ethical approval was not required for the studies on humans in accordance with the local legislation and institutional requirements because only commercially available established cell lines were used. Ethical approval was not required for the studies on animals in accordance with the local legislation and institutional requirements because only commercially available established cell lines were used.

## Author contributions

PL and FB-T conceived and planned the experiments. PL, VP, RM and LG carried out the experiments. PL wrote the manuscript with support from VP, RM, LG, FG and FB-T. All authors discussed the results and contributed to the final manuscript.
