# Supplementary figures and images for: Eotaxin-1/CCL11 promotes cellular senescence in human-derived fibroblasts through pro-oxidant and pro-inflammatory pathways
Source: Front Immunol. 2023 Oct 4;14:1243537. doi: 10.3389/fimmu.2023.1243537 (PMC10582634; doi:10.3389/fimmu.2023.1243537)

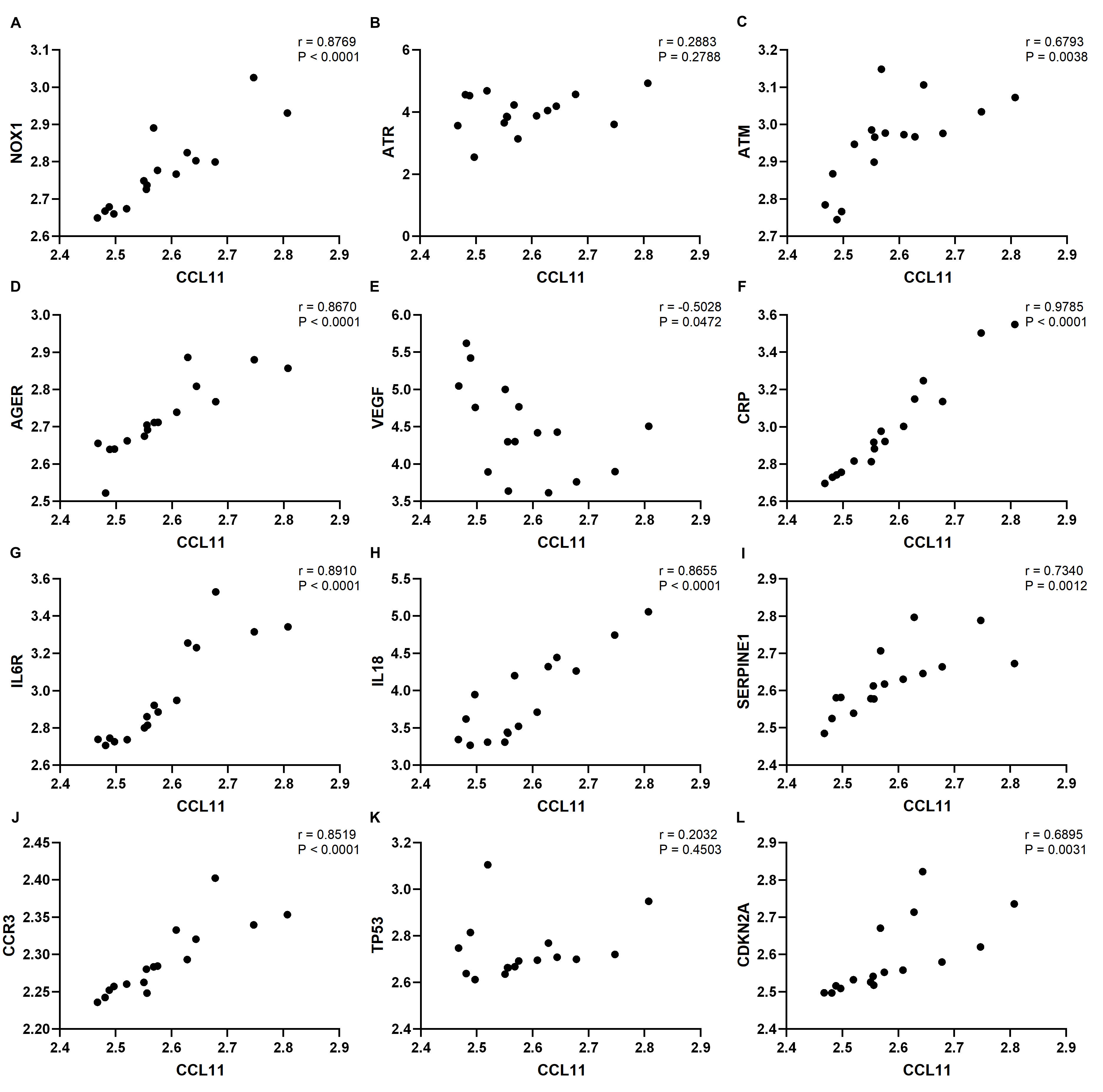

Supplement: Supplementary file 1 [file Image_1.tif]
